# Free Flap Reconstruction for Burn Injuries: A Single-Center Retrospective Comparison of Primary and Secondary Cases

**DOI:** 10.3390/jcm15176600

**Published:** 2026-08-26

**Authors:** Daihun Kang, Hyungwoo Wang, Seung Eun Hong, Youn Hwan Kim

**Affiliations:** 1 Department of Plastic and Reconstructive Surgery, Ewha Womans University Seoul Hospital, 260 Gonghang-daero, Gangseo-gu, Seoul 07804, Republic of Korea; gpk1234567@ewha.ac.kr; 2 College of Medicine, Ewha Womans University, Seoul 07804, Republic of Korea; 3 Department of Plastic and Reconstructive Surgery, Hanyang University Seoul Hospital, 222-1 Wangsimni-ro, Seongdong-gu, Seoul 04763, Republic of Korea; georgewang1996@gmail.com; 4 College of Medicine, Hanyang University, Seoul 04763, Republic of Korea

**Keywords:** burn reconstruction, free tissue transfer, thoracodorsal vessel, primary reconstruction, secondary reconstruction

## Abstract

**Background:** Most acute burn wounds are managed with skin grafting, yet many patients later develop contractures requiring secondary free flap reconstruction, which is widely believed to be more demanding than reconstruction in the acute setting. This clinical impression has rarely been quantified. This study compared the characteristics, operative parameters, and outcomes of primary and secondary free flap reconstruction for burn injuries. **Methods:** In this single-center retrospective study, 65 consecutive free flap reconstructions performed in 55 patients between 2012 and 2025 were categorized as primary (acute wound coverage during the initial burn admission, *n* = 35) or secondary (reconstruction of established scar contracture after discharge from acute care, *n* = 30). Demographics, burn extent, injury-to-reconstruction interval, prior operations, flap selection, operative time, secondary procedures, and complications were compared. Multivariable linear regression with sequential adjustment for age, sex, defect location, defect size, total body surface area (TBSA) burned, and flap complexity was used to estimate the adjusted difference in operative time. **Results:** Secondary patients were younger (40.8 vs. 55.1 years), more often female (70% vs. 29%), had larger burns (median TBSA 18% vs. 3%) and larger defects (348.5 vs. 203.3 cm^2^), and underwent reconstruction a median of 534 days after injury versus 30 days in the primary group. Operative time was longer in secondary cases (236.3 vs. 170.7 min; *p* < 0.001), and this association persisted after adjustment for age, sex, defect location, defect size, TBSA, and flap complexity (adjusted difference, 55.2 min; 95% CI, 20.4–90.1; *p* = 0.002) and in sensitivity analyses accounting for repeated procedures in the same patient. Flap complexity distribution and the frequency of secondary procedures did not differ between groups. Thoracodorsal vessel-based flaps were used in 90.8% of cases, and overall flap survival was 98.5%. **Conclusions:** Secondary free flap reconstruction for burn scar contracture required approximately 55 min longer than primary reconstruction of acute burn wounds, independently of defect size, burn extent, and flap complexity, whereas flap survival and the need for subsequent procedures were similar. Whether earlier free flap coverage of wounds at high risk of contracture could reduce this burden warrants prospective evaluation.

## 1. Introduction

Burns remain a leading cause of traumatic injury worldwide [1]. In the acute setting, the majority of burn wounds are managed with split-thickness skin grafts (STSG), and free flap reconstruction is reserved for wounds in which extensive soft tissue loss exposes bone, tendon, joint, or neurovascular structures that a graft cannot reliably cover [2]. A substantial proportion of grafted patients subsequently develop scar contractures that impair function and quality of life, and when local options are exhausted, free flap reconstruction is again required to achieve adequate release and durable resurfacing [3,4]. Free flap reconstruction after burns therefore occurs in two distinct clinical settings—primary coverage of the acute wound and secondary correction of established contracture—that differ in the condition of the recipient site, the antecedent treatment, and the goals of surgery.

Reconstructive surgeons widely assume that free flap reconstruction in previously burned and grafted tissue is more demanding than reconstruction in the acute setting, because exposure proceeds through scarred planes, recipient vessels must be prepared in a fibrotic field, and thorough scar excision and contracture release are required. This impression, however, has rarely been quantified, and it is unknown whether any such difference is explained by measurable factors—larger defects after contracture release, more extensive burns, or more complex flap configurations—or whether it reflects the operative environment itself. Answering this question requires a series that includes both primary and secondary reconstruction performed under comparable conditions.

A further consideration in burn patients is donor site availability. The anterolateral thigh (ALT) flap has traditionally served as a workhorse for soft tissue reconstruction [5,6], but in burn patients the thigh is frequently compromised, either because it lies within the burned area or because it has been used for skin graft harvest; a healed graft donor site does not in itself preclude flap harvest, but extensive lower-extremity burns and repeated grafting often limit the thigh in this population [7]. The thoracodorsal vascular system offers a compelling alternative [8]: it provides a long and reliable pedicle, large skin paddles, and the flexibility to create chimeric and linked configurations for complex three-dimensional defects [4,9,10], and the lateral trunk and back are less frequently burned and less commonly used for graft harvest, so this donor site is more likely to remain available [11]. At this institution, thoracodorsal vessel-based flaps have accordingly become the preferred option for burn reconstruction in both the acute and the delayed setting.

A previous study from this institution reported favorable outcomes with thin thoracodorsal artery perforator (TDAP) flaps for secondary burn scar contracture in 25 patients [4]. That report was confined to a single flap type and to secondary reconstruction. Since then, the surgical repertoire has expanded to include muscle-sparing, myocutaneous, chimeric, and linked configurations, and free flap reconstruction has been increasingly applied in the primary burn setting as well. The institutional experience—all flap types, in both primary and secondary reconstruction, performed by a single surgeon over 13 years —therefore provides an opportunity to compare the two clinical settings directly.

The present study analyzed this experience with two aims: first, to determine whether secondary reconstruction is associated with a greater operative burden than primary reconstruction after accounting for defect size, burn extent and flap complexity, and to characterize the overall surgical burden of each approach; and second, to describe the role and outcomes of thoracodorsal vessel-based flaps across both settings.

## 2. Methods

### 2.1. Study Design and Patient Selection

A retrospective review was conducted of patients who underwent free flap reconstruction for burn-related injuries at a single tertiary center between January 2012 and April 2025. The primary outcome was operative time, compared between primary and secondary reconstruction with adjustment for baseline differences; secondary outcomes were flap survival, complications, secondary procedures after the index flap, and the distribution and outcomes of flap types. Because operative time was the primary outcome, only procedures for which operative time was documented in the medical records were included; earlier cases in which operative time had not been recorded in a retrievable form were excluded. The study was approved by the Institutional Review Board of Hanyang University Seoul Hospital (IRB No. HYUH 2026-01-040; approved on 3 February 2026). Given the retrospective nature of the study, the requirement for informed consent was waived.

Four patients from the secondary group, treated between 2016 and 2018 for extremity contracture with TDAP flaps, were also included in the previous report from this institution [4]; the remaining patients of that report were treated before operative time was routinely documented and were therefore not eligible for the present analysis. That study did not include primary reconstruction cases and did not analyze operative time; the present primary cohort and the comparative, operative-time analysis are entirely new, and no outcome figure or table is reproduced from the earlier work.

Procedures were divided into two groups according to reconstruction timing. Primary reconstruction was defined as free flap coverage performed during the same hospital admission as the initial burn injury, before wound maturation, for an acute or subacute wound that had not healed with conservative measures or grafting; in this series, all primary reconstructions were performed within 12 months of injury, and 34 of 35 within 100 days. Secondary reconstruction was defined as free flap reconstruction performed after discharge from initial burn care for correction of an established scar contracture, unstable scar, or deformity.

### 2.2. Indications for Free Flap Reconstruction

In the primary setting, free flap reconstruction was indicated when a full-thickness burn wound, after adequate debridement, exposed bone, joint, tendon, or neurovascular structures that would not reliably support a skin graft, or when a chronic non-healing wound persisted despite conservative treatment; skin grafting alone was the initial treatment for all other acute burn wounds. In the secondary setting, free flap reconstruction was indicated for established scar contracture causing functional limitation, unstable or ulcerating scar, or deformity in which the required release created a defect too large for local flaps or in which skin grafting was expected to recontract, particularly across joints or in the neck.

### 2.3. Surgical Technique

All procedures were performed by a single senior surgeon (Y.H.K.). The surgical approach was tailored to individual defect characteristics, with three levels of complexity: (1) single flap (isolated free flap transfer, e.g., thoracodorsal artery perforator [TDAP], anterolateral thigh [ALT], or deep inferior epigastric perforator [DIEP] flap); (2) flap with graft (free flap combined with skin grafting); and (3) chimeric or linked flaps (multiple flap components based on the thoracodorsal vascular tree or combined with other donor sites for complex, extensive defects).

Flap selection was based on defect size, location, recipient vessel availability, and donor site condition. In patients where the thigh donor site was previously grafted or compromised, thoracodorsal vessel-based flaps were preferentially selected.

### 2.4. Data Collection

The following variables were extracted from medical records: demographics (age, sex, body mass index), burn etiology, total body surface area (TBSA) burned at the initial injury, burn depth (primary group), date of injury, operations performed before the free flap (debridement, skin grafting, previous flaps), defect location and size, flap type, recipient vessels, operative time, length of the hospital stay during which the free flap was performed, complications, secondary procedures performed after the free flap (debulking, scar revision, contracture release, additional grafting, flap division), and follow-up duration. For patients who underwent staged free flaps during a single course of treatment, subsequent procedures were attributed to the final index flap. Defect size was calculated as the product of the two largest perpendicular dimensions of the final defect after debridement or contracture release; for linked flaps covering more than one defect, areas were summed. The injury-to-reconstruction interval was calculated from the date of injury to the date of the free flap; when the injury date was documented only to the month or year, the mid-point of that period was used, and intervals are therefore reported as medians.

Operative time was defined as the total time from the first skin incision to completion of wound closure. It therefore included, as applicable, final debridement or scar excision and contracture release, recipient vessel exposure and preparation, flap harvest, microvascular anastomosis, flap inset, any concurrent skin grafting, and donor site closure. Time for anesthetic induction and positioning was not included. Because the durations of individual procedural phases were not recorded, only total operative time was available for analysis. Complications were classified as major (total flap loss) or minor (partial loss, hematoma, infection).

### 2.5. Statistical Analysis

Continuous variables are expressed as mean ± standard deviation and compared with independent *t*-tests, or, for skewed distributions (TBSA, injury-to-reconstruction interval, length of stay, follow-up, secondary procedure counts), as median [interquartile range] and compared with the Mann–Whitney U test. Categorical variables were analyzed with chi-square or Fisher’s exact tests as appropriate. Effect sizes were calculated as Cohen’s d for continuous variables and odds ratios for categorical variables, with 95% confidence intervals (CI).

One-way analysis of variance with post-hoc pairwise *t*-tests was used to compare operative times across flap complexity categories. Flap survival was calculated with an exact binomial 95% CI. To compare operative time between reconstruction groups while accounting for baseline imbalances, multivariable linear regression models were fitted with operative time as the dependent variable and reconstruction timing (secondary versus primary) as the exposure. Covariates were added sequentially: Model 1 included age, sex, and defect location; Model 2 added flap complexity; Model 3 added defect size to Model 1; Model 4 added defect size and TBSA to Model 1; and Model 5, the pre-specified primary model, included age, sex, defect location, defect size, TBSA, and flap complexity. Case year was added in a further sensitivity model to assess a possible effect of surgical experience over the study period. Because six patients contributed more than one flap, standard errors clustered by patient were computed for every model, and a sensitivity analysis restricted to the first flap in each patient was performed. Adjusted mean differences with 95% CIs are reported. Baseline comparisons were exploratory and were not corrected for multiple testing. Multivariable modeling of complications was not undertaken because the small number of events would risk overfitting.

Statistical significance was set at *p* < 0.05. All analyses were performed using Python 3.11 (SciPy 1.11, statsmodels 0.14).

## 3. Results

### 3.1. Patient Demographics

A total of 65 free flap procedures were performed in 55 patients (30 men, 25 women) during the study period. Six patients underwent more than one free flap (two flaps in four patients and four flaps in two patients), in all cases for separate defects or staged reconstruction. Mean age at operation was 48.5 ± 18.7 years (range, 11–85 years). The primary group comprised 35 procedures in 30 patients and the secondary group 30 procedures in 25 patients; no patient contributed procedures to both groups. The most common etiologies were friction burns (*n* = 17, 26.2%), flame burns (*n* = 15, 23.1%), scald burns (*n* = 12, 18.5%), and contact burns (*n* = 10, 15.4%), with the remainder comprising electrical injury (*n* = 5), cold injury (*n* = 4), radiation injury (*n* = 1), and chemical burn (*n* = 1). All primary reconstructions were performed for third-degree (*n* = 31) or fourth-degree (*n* = 4) burns. Median follow-up was 7 [3–17] months (range, 0–48), with no significant difference between groups (Table 1).

### 3.2. Comparison Between Primary and Secondary Reconstruction Groups

Baseline characteristics are summarized in Table 1. Patients undergoing secondary reconstruction were significantly younger than those undergoing primary reconstruction (40.8 ± 17.8 vs. 55.1 ± 17.0 years; mean difference, 14.3 years; 95% CI, 5.7–23.0; *p* = 0.002; Cohen’s d = 0.83), and a higher proportion were female (70% vs. 29%; odds ratio, 5.83; 95% CI, 2.0–17.0; *p* = 0.001).

The two groups differed markedly in the timing and antecedents of reconstruction. In the primary group, free flap coverage was performed a median of 30 [22–52] days after injury (range, 9–334 days), whereas in the secondary group, the median interval was 534 [377–1296] days (approximately 1.5 [1.0–3.5] years; range, 94 days to 44 years). The intervals of the two groups overlapped at their extremes—one primary reconstruction was performed at 334 days for a persistently unhealed wound, and one secondary reconstruction at 94 days for an early contracture—reflecting that group assignment was based on the clinical status of the wound rather than on a fixed time threshold. Before the free flap, 33 of 35 primary procedures (94%) had been preceded by debridement only, whereas 26 of 30 secondary procedures (87%) were performed in previously skin-grafted fields and 2 (7%) in fields previously reconstructed with a free flap.

The extent of the initial injury and the size of the reconstructed defect also differed. Secondary patients had sustained more extensive burns at the initial injury (median TBSA, 18% [3–27] vs. 3% [2–11]; *p* = 0.002), whereas primary patients had typically sustained small but deep injuries exposing critical structures. The defects ultimately reconstructed with a free flap were nonetheless larger in the secondary group (348.5 ± 249.5 vs. 203.3 ± 165.4 cm^2^; *p* = 0.007; Cohen’s d = 0.70), reflecting the extent of resurfacing required after contracture release. Defect location differed as well (*p* = 0.008), with upper-extremity defects predominating among primary and head-and-neck and trunk defects among secondary reconstructions. The distribution of flap complexity did not differ between groups (*p* = 0.44).

Mean operative time was significantly longer in the secondary group (236.3 ± 88.1 vs. 170.7 ± 50.3 min; mean difference, 65.6 min; 95% CI, 30.7–100.5; *p* < 0.001; Cohen’s d = 0.93). Length of the index hospital stay was longer in the primary group (median, 40 [33–75] vs. 28 [19–37] days; *p* < 0.001), reflecting the inclusion of acute burn care in the same admission.

### 3.3. Flap Selection and Complexity

Operative time differed across flap complexity categories (Table 2): single flap (*n* = 37, 56.9%; 185.3 ± 76.2 min), flap with graft (*n* = 18, 27.7%; 182.2 ± 34.6 min), and chimeric or linked flaps (*n* = 10, 15.4%; 293.0 ± 75.2 min; ANOVA F = 11.08, *p* < 0.001). Chimeric or linked flaps required longer operative times than both single flaps (*p* < 0.001) and flap-with-graft procedures (*p* < 0.001), whereas single flaps and flap-with-graft procedures did not differ (*p* = 0.87). As noted above, the distribution of complexity did not differ between primary and secondary groups.

Thoracodorsal vessel-based flaps were used in 59 of 65 procedures (90.8%), including TDAP flaps, latissimus dorsi muscle-sparing flaps, and latissimus dorsi myocutaneous flaps, alone or in linked configurations (Table 3). The remaining procedures used ALT flaps alone (*n* = 3), DIEP flaps alone (*n* = 2), or an ALT flap with STSG (*n* = 1). Four of the six non-thoracodorsal procedures were performed in patients who had previously received thoracodorsal-based flaps, indicating that alternative donor sites were selected primarily when the thoracodorsal system was no longer available.

### 3.4. Multivariable Analysis of Operative Time

Results of the sequential regression models are presented in Table 4. After adjustment for age, sex, and defect location (Model 1), secondary reconstruction remained associated with a longer operative time (adjusted mean difference, 57.2 min; 95% CI, 14.4–99.9; *p* = 0.010). Because defects were larger and burns more extensive in the secondary group, defect size and TBSA were added as covariates. Adjustment for defect size alone attenuated the estimate (Model 3: 39.5 min; 95% CI, 1.1–77.9; *p* = 0.044), and adjustment for defect size and TBSA together yielded an adjusted difference of 45.7 min (95% CI, 8.4–83.0; *p* = 0.017). In the primary model additionally including flap complexity (Model 5; R^2^ = 0.59), secondary reconstruction was associated with an adjusted difference of 55.2 min (95% CI, 20.4–90.1; *p* = 0.002). In this model, larger defect size (12.0 min per 100 cm^2^; *p* = 0.006) and chimeric or linked configuration (*p* = 0.001) were independently associated with longer operative time, whereas greater TBSA was associated with shorter operative time (−1.7 min per 1% TBSA; *p* = 0.006); age and sex were not significant.

The estimate was stable in sensitivity analyses. Standard errors clustered by patient gave a 95% CI of 22.0–88.4 min (*p* = 0.001) for the primary model, and restriction to the first flap in each patient (*n* = 55) yielded an adjusted difference of 58.6 min (95% CI, 18.1–99.2; *p* = 0.006). Case year was similarly distributed between groups (median 2021 in both; *p* = 0.91), and its inclusion did not alter the estimate (55.0 min; 95% CI, 19.8–90.1; *p* = 0.003), arguing against confounding by evolving surgical experience.

### 3.5. Complications and Flap Survival

Three complications occurred in 65 procedures (4.6%): one hematoma (primary group, TDAP flap), one total flap loss due to arterial insufficiency (secondary group, latissimus dorsi muscle-sparing flap), and one partial loss of the distal tip (secondary group, TDAP flap with STSG). Overall flap survival was 98.5% (64/65; 95% CI, 91.7–100%). Complication rates did not differ between primary and secondary groups (2.9% vs. 6.7%; *p* = 0.59).

### 3.6. Secondary Procedures and Overall Surgical Burden

Secondary procedures after the index free flap are summarized in Table 5. At least one secondary procedure was performed after 14 of 35 primary flaps (40%) and 16 of 30 secondary flaps (53%; *p* = 0.33); the median number of secondary procedures per flap was 0 [0–1] and 1 [0–2], respectively (*p* = 0.20). Debulking was the most frequent secondary procedure (31% of primary and 50% of secondary flaps; *p* = 0.14), followed by scar revision or contracture release (6% vs. 17%; *p* = 0.23). On a per-patient basis, the number of free flaps (1.2 ± 0.6 in both groups) and the proportion of patients requiring any secondary procedure (47% vs. 56%; *p* = 0.59) were similar.

### 3.7. Illustrative Cases

**Figure 1:**
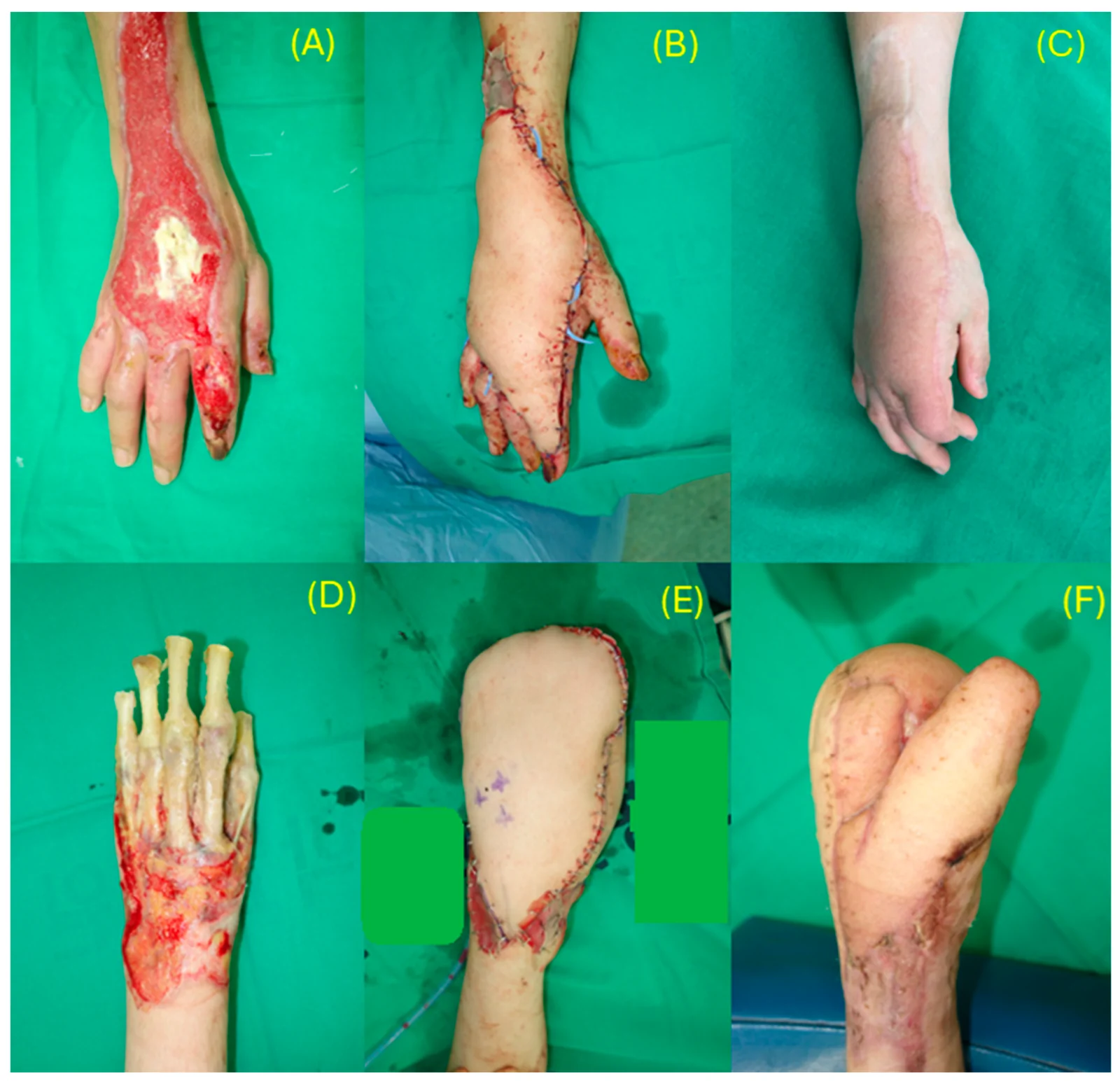
Primary free flap reconstruction of acute burn wounds. (**A**) A 50-year-old woman with a contact burn to the dorsum of the hand with exposed extensor tendons and second-finger joint. (**B**) Immediate postoperative view after coverage with a 22 × 8 cm thoracodorsal artery perforator (TDAP) flap. (**C**) Result at 3 years 6 months, after one debulking and web-space release with full-thickness skin grafting. (**D**) A 27-year-old woman with a severe flame burn to the hand with exposed bone, for which amputation had been recommended elsewhere. (**E**) Immediate postoperative view after debridement and reconstruction with a TDAP–anterolateral thigh linked flap to create a mitten hand. (**F**) Result at 3 months, after thumb separation.

**Figure 2:**
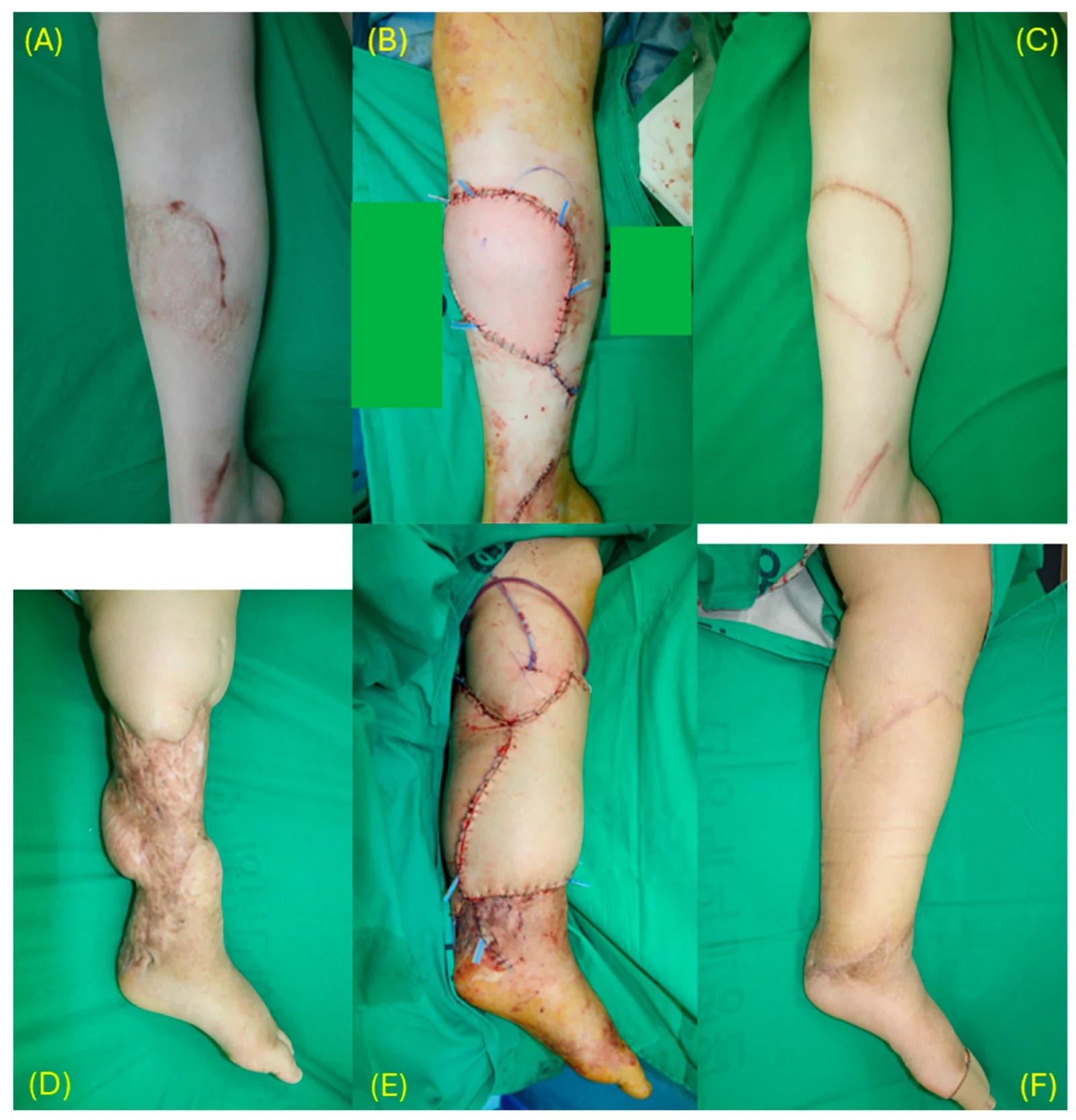
Secondary free flap reconstruction of burn scar contracture. (**A**) A 20-year-old woman with contracture and unstable scar of the lower leg after split-thickness skin grafting for a friction burn. (**B**) Immediate postoperative view after contracture release and resurfacing with a TDAP flap. (**C**) Result at 2 years, after two debulking procedures and scar revision. (**D**) A 28-year-old woman with severe scarring and contracture of the lower leg and ankle after a previous latissimus dorsi muscle flap and skin graft for a friction burn. (**E**) Immediate postoperative view after scar excision, contracture release, and reconstruction with a latissimus dorsi muscle-sparing flap combined with a deep inferior epigastric perforator flap. (**F**) Result at 2 years, after two debulking procedures and scar revision.

Representative cases from each reconstruction group are presented in Figure 1 and Figure 2.

A 50-year-old female patient presented with a thermal contact burn to the dorsum of the hand with exposed extensor tendons and second finger joint (Figure 1A–C). Reconstruction was performed using a 22 × 8 cm TDAP flap. One session of debulking surgery and web space contracture release with full-thickness skin grafting were subsequently performed. The photographs were taken 3 years and 6 months postoperatively. A 27-year-old female patient sustained a severe flame burn to the hand (Figure 1D–F). Amputation was recommended at another hospital; however, in an effort to preserve the limb, thorough debridement was performed followed by reconstruction using a TDAP-ALT linked flap to create a mitten hand. The photographs were taken 3 months postoperatively.

A 20-year-old female patient with bilateral lower extremity contractures and scarring following split-thickness skin grafting for friction burns underwent resurfacing with a TDAP flap (Figure 2A–C). Two debulking procedures and scar revisions were performed. The 2-year postoperative outcome is shown. A 28-year-old female patient with severe scarring and contracture following previous latissimus dorsi muscle flap and split-thickness skin graft reconstruction for a friction burn underwent reconstruction using a latissimus dorsi muscle-sparing flap combined with a deep inferior epigastric perforator flap (Figure 2D–F). Two debulking procedures and scar revisions were performed. The 2-year postoperative result is shown.

## 4. Discussion

This study analyzed 65 consecutive free flap reconstructions for burn injuries performed in 55 patients over a 13-year period, extending the previously reported institutional experience with TDAP flaps for secondary burn scar contracture [4]. Three principal findings emerged. First, primary and secondary reconstruction served distinct patient populations: primary flaps were used for older patients with small, deep injuries and exposed critical structures, whereas secondary flaps were used for younger, predominantly female patients with more extensive burns who presented a median of 1.5 years after injury with established contracture in previously grafted fields. Second, secondary reconstruction was associated with an operative time approximately 55 min longer than primary reconstruction, an association that persisted after adjustment for age, sex, defect location, defect size, TBSA, and flap complexity and in sensitivity analyses accounting for repeated procedures. Third, thoracodorsal vessel-based flaps were used in the vast majority of cases in both settings with a flap survival rate of 98.5%, and the frequency of subsequent secondary procedures did not differ between groups.

### 4.1. Two Distinct Populations

The demographic and injury profiles of the two groups illustrate the different clinical pathways that lead to free flap reconstruction after burns. Secondary reconstruction was performed in younger patients, more often women, who had survived larger burns (median TBSA 18%) managed initially with skin grafting and who later sought correction of contracture; younger patients and women may be more strongly motivated to seek correction of burn sequelae for functional and aesthetic reasons, as burn scars are known to affect quality of life through appearance-related concerns and psychosocial distress [12,13]. Primary reconstruction, by contrast, was performed predominantly in older patients with limited TBSA (median 3%) but full-thickness injury exposing bone, tendon, or joint, most often on the hand or lower leg; the predominance of older patients likely reflects their vulnerability to deep injury from thinner skin, diminished reaction times, and comorbidity [14,15]. These differences underscore that the present comparison is between two populations and two indications, not between two strategies applied to the same wound.

### 4.2. Operative Time in Secondary Reconstruction

The longer operative time in secondary cases (236 vs. 171 min) is the central finding of this study. Flap complexity did not differ between groups, and adjustment for it did not attenuate the association. Defects were larger in the secondary group, and adjustment for defect size reduced the estimate, indicating that part of the crude difference reflects the greater extent of resurfacing required after contracture release; nevertheless, a difference of approximately 40–55 min persisted after adjustment for defect size, TBSA, flap complexity, and case year. The inverse association between TBSA and operative time in the full model reflects the profile of the primary group, in which small but deep injuries required flap coverage, rather than any technical simplicity of larger burns.

The remaining difference is plausibly related to the operative environment of secondary reconstruction—exposure through scarred planes, recipient vessel preparation in a fibrotic field, and thorough scar excision and contracture release [16,17]. Because only total operative time was recorded, the relative contribution of these components cannot be separated here; contracture release alone is a substantive step not required in primary cases and may account for much of the difference. What the present data do establish is that the additional hour of secondary reconstruction is not an artifact of larger defects, more extensive burns, or more complex flaps—the explanations most readily invoked—but is intrinsic to operating in a previously burned and grafted field, whatever its precise mechanism. To the authors’ knowledge, this is the first study to quantify that burden and to test these alternative explanations directly, and the estimate of roughly 55 min provides both a concrete figure for preoperative counseling and operating room planning and an effect size on which prospective studies can be powered. Such studies could resolve the mechanism by logging procedural phases separately—debridement or contracture release, recipient vessel preparation, flap harvest, anastomosis, and inset—and combining phase-specific timing with grading of recipient vessel quality and scar fibrosis. Nor is the additional burden of secondary reconstruction spread across the treatment course: the number of secondary procedures after the index flap, the proportion of flaps requiring debulking or scar revision, and the number of free flaps per patient were similar in the two groups, indicating that once free flap reconstruction is undertaken, the downstream refinement burden is comparable regardless of timing and the difference is concentrated in the index operation. Length of stay was longer in the primary group only because the same admission encompassed acute burn care.

### 4.3. Outcomes

The overall flap survival rate of 98.5% (64/65) is comparable to previously reported outcomes in burn free flap reconstruction. Jabir et al. reported 100% flap survival with a mixed flap approach in 23 patients [18], Brewin et al. reported 97.8% survival (45/46 flaps) [19], and Carolina et al. reported favorable outcomes utilizing ALT flaps in 81% of their 36 cases [20]. Ziegler et al., reviewing free flaps in acute and reconstructive burn care, reported a 7% flap loss rate among 14 acute flaps and lower complication rates in the reconstructive setting [21]. A recent systematic review of 396 microsurgical burn reconstructions reported mean flap success rates of 92.7% for acute and 95.7% for delayed reconstruction [22], and a meta-analysis of delayed burn reconstruction reported pooled rates of flap failure and contracture recurrence that were low but not negligible [7]. Timing within the acute period is itself a determinant of outcome: a meta-analysis of 275 acute burn free flaps found the highest failure rate for flaps performed 5–21 days after injury (16.6%), compared with 7.3% within 4 days and 6.7% between 22 days and 6 weeks [23]; in the present series, most primary flaps were performed beyond this window (median 30 days). Notably, the high success rate in the present series was maintained in both primary and secondary reconstruction groups despite the greater operative demands of the latter. The single total flap loss occurred in a secondary case due to arterial insufficiency, highlighting the importance of thorough preoperative vascular assessment and alternative recipient vessel planning when operating in scarred fields.

### 4.4. The Question of Earlier Intervention

The current standard of care is to cover acute burn wounds with skin grafts and to reserve free flap reconstruction for the contractures that later develop; primary free flap coverage is undertaken only when critical structures are exposed. This sequence rests on the reasonable premise that most grafted wounds heal without severe contracture [3]. Yet the corollary—that the free flap performed later, when a contracture has formed, is a materially different and more demanding operation than the one that might have been performed at the outset—has largely remained a clinical impression. The present data give that impression a measurable form: when secondary free flap reconstruction does become necessary, the index operation is approximately 55 min longer, and this difference is not accounted for by defect size, burn extent, or flap complexity. Together with the high flap survival achieved in primary cases, this observation makes it legitimate to ask whether, for burn wounds in which contracture is highly predictable—full-thickness injuries crossing the neck, axilla, popliteal fossa, or hand—earlier definitive coverage with a free flap might in selected patients spare a later and more demanding operation. Early definitive coverage with a free flap is already accepted practice in selected chemical injuries, such as hydrofluoric acid burns of the hand, in which progressive tissue necrosis favors early radical debridement and flap reconstruction over expectant management [24]. This study raises that question; it does not answer it, and the question is offered here strictly as a hypothesis for prospective testing rather than as a recommendation to alter current practice.

An essential caveat qualifies this observation. This study compared patients who underwent primary free flaps with those who underwent secondary free flaps—two distinct populations with different injuries and indications—rather than comparing early free flap coverage with skin grafting followed by later flap reconstruction. Critically, it does not capture the far larger group of patients whose acute burns were managed with skin grafting and who never required flap surgery. Expanding indications for primary free flaps would necessarily entail microsurgery in some patients who would have healed satisfactorily with grafting alone, a trade-off that these data cannot quantify, and any such shift would also have to respect the timing-related risk of flap loss in the acute setting [23]. The finding that secondary reconstruction requires longer operations therefore raises, but cannot answer, the question of whether earlier intervention is warranted. A definitive answer requires a prospective comparison of early free flap coverage versus initial skin grafting with selective later reconstruction, using long-term functional outcomes, contracture recurrence, and total surgical burden as endpoints.

### 4.5. Thoracodorsal Vessel Predominance in Burn Reconstruction

Thoracodorsal vessel-based flaps were used in 90.8% of cases. In secondary reconstruction, the high utilization of thoracodorsal-based flaps is largely driven by donor site constraints. Epidemiological studies have consistently shown that the extremities are the most common sites of burn injury [25]. Because extremity burns frequently require STSG for acute wound coverage, the thigh—the typical ALT flap donor site—is often unavailable for subsequent flap harvest due to prior skin graft harvest or direct burn involvement [7]. In contrast, the lateral trunk and back are less frequently burned and less commonly used for skin graft harvest, leaving the thoracodorsal region available as a donor site [11].

In primary reconstruction, where the thigh may still be available, the preference for thoracodorsal-based flaps in this series primarily reflects the senior surgeon’s familiarity and extensive experience with this vascular system. It should be acknowledged that flap selection was based on surgeon preference and clinical judgment rather than a standardized protocol, and the relative merits of thoracodorsal-based versus ALT-based approaches in primary burn reconstruction cannot be determined from this series.

Regardless of the reason for selection, the thoracodorsal vascular system offers inherent versatility for burn reconstruction. From simple TDAP perforator flaps for moderate defects to complex chimeric and linked configurations incorporating muscle-sparing or myocutaneous components, the system can be tailored to diverse defect requirements through a single donor site [4,9,10]. However, this approach has its limits—four of the six non-thoracodorsal flap cases occurred in patients whose thoracodorsal system had already been used in a prior reconstruction. This highlights the importance of planning the long-term reconstructive sequence in burn patients, as exhaustion of the thoracodorsal donor site necessitates alternative options such as ALT or DIEP flaps.

### 4.6. Limitations

This study has several limitations. As a retrospective, single-center series performed by a single surgical team, the findings reflect specific practice patterns and may not generalize. Most importantly, the design compares two different patient populations—primary versus secondary flap recipients—with different injuries, indications, and antecedent treatment, and includes no skin-graft-only reference group; it therefore cannot establish whether earlier free flap reconstruction reduces the overall need for, or burden of, later surgery, and the discussion of expanded primary indications should be regarded as hypothesis-generating. Although defect size, TBSA, prior operations, and flap complexity were accounted for, residual confounding by unmeasured factors such as recipient vessel condition, scar quality, and the extent of contracture release cannot be excluded. Only total operative time was recorded; step-specific times were not available, so the attribution of the additional operative time to any particular component of secondary reconstruction remains inferential. Operative time is an objective and reproducible measure of surgical demand but a surrogate endpoint that does not directly capture patient-centered benefit, and patient-reported outcome measures was not collected. Injury dates were documented only to the month or year in a proportion of secondary cases, so the injury-to-reconstruction interval is reported as a median. Follow-up was short and variable (median 7 months) for assessing long-term function and contracture recurrence, and secondary-procedure counts may be incomplete for patients followed elsewhere. Because a single surgeon accrued cases over a prolonged period, evolving experience might have influenced operative times; however, case year was similarly distributed between groups and did not alter the principal findings. Six patients contributed more than one flap; cluster-robust standard errors and a first-flap-only sensitivity analysis yielded consistent results. Baseline comparisons were exploratory and not corrected for multiple testing. The small number of complications precluded multivariable modeling of risk factors. Because inclusion required documented operative time, earlier cases at this institution were under-represented, and the series should not be regarded as a complete census of burn free flap reconstruction over the study period; this restriction was applied uniformly to both groups and, since case year did not differ between them, is unlikely to have biased the comparison. Finally, a small subset of the secondary group was included in a previous report from this institution, as disclosed in the Methods.

## 5. Conclusions

In this 13-year single-center series of 65 free flap reconstructions in 55 burn patients, primary and secondary reconstruction served distinct populations: primary flaps were used for older patients with small, deep injuries exposing critical structures; and secondary flaps for younger, predominantly female patients with more extensive burns who presented a median of 1.5 years after injury with established contracture in previously grafted fields. Secondary reconstruction was associated with an operative time approximately 55 min longer than primary reconstruction, and this association persisted after adjustment for age, sex, defect location, defect size, TBSA, and flap complexity and in sensitivity analyses—indicating that the additional burden is intrinsic to operating in a previously burned and grafted field rather than an artifact of larger defects, more extensive burns, or more complex flaps. Flap survival (98.5%) and the frequency of subsequent secondary procedures did not differ between groups, so the additional burden of secondary reconstruction is concentrated in the index operation. Thoracodorsal vessel-based flaps, used in over 90% of cases, proved reliable in both settings.

Because the study compares two distinct patient populations and lacks a skin-graft-only reference group, it cannot establish whether earlier free flap coverage would reduce the overall surgical burden of burn reconstruction. The estimate reported here provides a concrete figure for preoperative counseling and operating room planning and an effect size for prospective comparison of early free flap coverage against initial skin grafting with selective later reconstruction, using phase-specific operative timing, long-term functional outcomes, and total surgical burden as endpoints.

## Figures and Tables

**Table 1:** Comparison of Primary and Secondary Reconstruction Groups.

Variable	Primary (*n* = 35)	Secondary (*n* = 30)	*p*-Value	Effect Size
Age (years)	55.1 ± 17.0	40.8 ± 17.8	0.002	d = 0.83
Sex, female, *n* (%)	10 (28.6)	21 (70.0)	0.001	OR = 5.83
BMI (kg/m^2^)	22.8 ± 3.2	23.6 ± 3.6	0.38	d = 0.24
TBSA burned (%)	3 [2–11]	18 [3–27]	0.002	–
Interval from injury to free flap (days)	30 [22–52]	534 [377–1296]	<0.001	–
Range	9–334	94–16,276		
Prior surgery before free flap, *n* (%)			<0.001	–
Debridement only	33 (94.3)	2 (6.7)		
Skin graft (STSG/FTSG)	0 (0)	26 (86.7)		
Previous free flap	0 (0)	2 (6.7)		
None	2 (5.7)	1 (3.3)		
Defect location, *n* (%)			0.008	–
Head & neck	1 (2.9)	4 (13.3)		
Upper extremity	13 (37.1)	3 (10.0)		
Lower extremity	21 (60.0)	19 (63.3)		
Trunk & axilla	0 (0)	4 (13.3)		
Defect size (cm^2^)	203.3 ± 165.4	348.5 ± 249.5	0.007	d = 0.70
Flap complexity, *n* (%)			0.44	–
Single flap	18 (51.4)	19 (63.3)		
Flap with graft	12 (34.3)	6 (20.0)		
Chimeric/linked	5 (14.3)	5 (16.7)		
Operative time (min)	170.7 ± 50.3	236.3 ± 88.1	<0.001	d = 0.93
Length of hospital stay (days) *	40 [33–75]	28 [19–37]	<0.001	–
Complications, *n* (%)	1 (2.9)	2 (6.7)	0.59	–
Follow-up (months)	7 [4–12]	8 [3–21]	0.58	–

Values are mean ± SD, median [interquartile range], or *n* (%). Continuous variables were compared with independent *t*-tests (mean ± SD) or Mann–Whitney U tests (median [IQR]); categorical variables with Fisher’s exact or chi-square tests. Effect sizes: Cohen’s d for continuous variables, odds ratio (OR) for sex. Injury dates were available to the day in 32/35 primary and 12/30 secondary cases, to the month in 3 and 8, and to the year only in 0 and 10, respectively; mid-point imputation was used for incomplete dates, and intervals are therefore reported as medians. * Length of stay refers to the admission during which the free flap was performed; in the primary group, this admission also encompassed acute burn care and is not directly comparable. BMI, body mass index; TBSA, total body surface area; STSG, split-thickness skin graft; FTSG, full-thickness skin graft.

**Table 2:** Operative Time by Flap Complexity.

Flap Category	*n* (%)	Operative Time (min)	*p*-Value vs. Chimeric/Linked
Single flap	37 (56.9)	185.3 ± 76.2	<0.001
Flap with graft	18 (27.7)	182.2 ± 34.6	<0.001
Chimeric/linked	10 (15.4)	293.0 ± 75.2	Reference

One-way ANOVA: F = 11.08, *p* < 0.001. Single flap vs. flap with graft: *p* = 0.87. Distribution of complexity did not differ between primary and secondary groups (chi-square *p* = 0.44).

**Table 3:** Flap Types Used.

Flap Type	Primary (*n* = 35)	Secondary (*n* = 30)	Total, *n* (%)
Thoracodorsal-based	33	26	59 (90.8)
TDAP alone	15	12	27
TDAP + STSG/FTSG	10	4	14
LD muscle-sparing	1	3	4
LDmc ± STSG	1	2	3
TDAP + ALT ± STSG (linked)	5	2	7
TDAP + DIEP (linked)	0	3	3
TDAP + local flap + STSG	1	0	1
Non-thoracodorsal	2	4	6 (9.2)
ALT alone	2	1	3
DIEP alone	0	2	2
ALT + STSG	0	1	1

TDAP, thoracodorsal artery perforator flap; LD, latissimus dorsi; LDmc, latissimus dorsi myocutaneous flap; ALT, anterolateral thigh flap; DIEP, deep inferior epigastric perforator flap; STSG, split-thickness skin graft; FTSG, full-thickness skin graft.

**Table 4:** Multivariable Linear Regression: Adjusted Difference in Operative Time (Secondary vs. Primary) Across Sequential Models.

Model (Covariates)	Adjusted Difference (min)	95% CI	*p*-Value	Cluster-Robust 95% CI; *p* *
Model 1: age, sex, defect location	57.2	14.4 to 99.9	0.010	15.3 to 99.0; 0.007
Model 2: Model 1 + flap complexity	59.7	23.5 to 95.9	0.002	25.2 to 94.2; 0.001
Model 3: Model 1 + defect size	39.5	1.1 to 77.9	0.044	4.0 to 75.0; 0.029
Model 4: Model 1 + defect size + TBSA	45.7	8.4 to 83.0	0.017	11.1 to 80.3; 0.010
Model 5 (primary model): Model 4 + flap complexity	55.2	20.4 to 90.1	0.002	22.0 to 88.4; 0.001
Model 6: Model 5 + case year	55.0	19.8 to 90.1	0.003	22.3 to 87.6; 0.001
Sensitivity: first flap per patient only (*n* = 55)				
Model 4 covariates	50.5	7.9 to 93.1	0.021	–
Model 5 covariates	58.6	18.1 to 99.2	0.006	–

Dependent variable: operative time (min), *n* = 65 flaps in 55 patients. In the primary model (Model 5; R^2^ = 0.59), defect size (+12.0 min per 100 cm^2^, *p* = 0.006) and chimeric/linked configuration (*p* = 0.001) were independently associated with longer operative time, whereas greater TBSA was associated with shorter operative time (−1.7 min per 1%, *p* = 0.006), consistent with the smaller, deeper injuries treated in the primary group; age and sex were not significant. * Standard errors clustered by patient to account for repeated procedures in the same individual. TBSA, total body surface area.

**Table 5:** Overall Surgical Burden and Secondary Procedures After Free Flap Reconstruction.

Variable	Primary	Secondary	*p*-Value
Per flap	(*n* = 35)	(*n* = 30)	
Any secondary procedure, *n* (%)	14 (40.0)	16 (53.3)	0.33
Number of secondary procedures per flap	0 [0–1]; 1.1 ± 2.2	1 [0–2]; 1.2 ± 1.5	0.20
Debulking, *n* (%)	11 (31.4)	15 (50.0)	0.14
Scar revision/contracture release, *n* (%)	2 (5.7)	5 (16.7)	0.23
Per patient	(*n* = 30)	(*n* = 25)	
Free flaps per patient	1.2 ± 0.6	1.2 ± 0.6	–
Patients with > 1 free flap, *n*	3	3	–
Any secondary procedure, *n* (%)	14 (46.7)	14 (56.0)	0.59
Secondary procedures per patient	1.2 ± 2.3	1.5 ± 1.8	–

Values are *n* (%), median [interquartile range], or mean ± SD. Secondary procedures comprised debulking, scar revision, Z-plasty, contracture release, additional skin grafting, and flap division performed after the index free flap. In one primary patient who underwent four staged free flaps during a single course of treatment, subsequent procedures were recorded once for the patient and attributed to the final index flap. Fisher’s exact test for proportions; Mann–Whitney U test for counts.

## Data Availability

The data presented in this study are available on request from the corresponding author. The data are not publicly available because they contain information that could compromise the privacy of research participants. The analysis script used to generate the results is likewise available from the corresponding author on request.
